# Chemokines in Gestational Diabetes Mellitus

**DOI:** 10.3389/fimmu.2022.705852

**Published:** 2022-02-08

**Authors:** Hongying Liu, Aizhong Liu, Atipatsa C. Kaminga, Judy McDonald, Shi Wu Wen, Xiongfeng Pan

**Affiliations:** ^1^ Department of Epidemiology and Health Statistics, Xiangya School of Public Health, Central South University, Changsha, China; ^2^ Hunan Provincial Key Laboratory of Clinical Epidemiology, Xiangya School of Public Health, Central South University, Changsha, China; ^3^ Department of Mathematics and Statistics, Mzuzu University, Mzuzu, Malawi; ^4^ McLaughlin Centre for Population Health Risk Assessment, Faculty of Medicine, University of Ottawa, Ottawa, ON, Canada; ^5^ OMNI Research Group, Ottawa Hospital Research Institute, Ottawa, ON, Canada; ^6^ Department of Obstetrics and Gynaecology and School of Epidemiology and Public Health, University of Ottawa Faculty of Medicine, Ottawa, ON, Canada

**Keywords:** chemokines, gestational diabetes mellitus, inflammatory, immune microenvironment, meta-analysis

## Abstract

**Background:**

Studies investigating chemokines in gestational diabetes mellitus (GDM) have yielded mixed results. The purpose of this meta-analysis was to explore whether concentrations of chemokines in patients with GDM differed from that of the controls.

**Methods:**

Following Preferred Reporting Items for Systematic Reviews and Meta-Analyses (PRISMA) guidelines, we systematically searched Web of Science, Embase, Cochrane Library, and PubMed databases for articles, published in any language, on chemokines and GDM through August 1st, 2021. The difference in concentrations of chemokines between patients with GDM and controls was determined by a standardized mean difference (SMD) with a 95% confidence interval (CI), calculated in the meta-analysis of the eligible studies using a random-effects model with restricted maximum-likelihood estimator.

**Results:**

Seventeen studies met the inclusion criteria for the meta-analysis. Altogether, they included nine different chemokines comparisons involving 5,158 participants (1,934 GDM patients and 3,224 controls). Results showed a significant increase of these chemokines (CCL2, CXCL1, CXCL8, CXCL9, and CXCL12) in the GDM patients compared with the controls. However, there was a significant decrease of the chemokines, CCL4, CCL11 and CXCL10, in the GDM patients compared with the controls. Moreover, subgroup analysis revealed a potential role of chemokines as biomarkers in relation to laboratory detection (different sample type and assay methods) and clinical characteristics of GDM patients (ethnicity and body mass index).

**Conclusion:**

GDM is associated with several chemokines (CCL2, CCL4, CCL11, CXCL1, CXCL8, CXCL9, CXCL10 and CXCL12). Therefore, consideration of these chemokines as potential targets or biomarkers in the pathophysiology of GDM development is necessary. Notably, the information of subgroup analysis underscores the importance of exploring putative mechanisms underlying this association, in order to develop new individualized clinical and therapeutic strategies.

## Introduction

In recent years, the incidence of gestational diabetes mellitus (GDM) has increased rapidly worldwide and constitutes a major public health problem (1). GDM leads to adverse short-term perinatal complications (e.g., eclampsia, preeclampsia, placental malfunction, diabetic fetopathy, and jaundice, etc.) and long-term metabolic disorders complications (e.g., increased risk of developing hypertension, Type 2 Diabetes Mellitus (T2DM), metabolic syndrome, and atherosclerotic, etc.) in both the offspring and mothers (2–4).

So far, GDM cannot be cured because its pathogenesis is not well understood (4). Therefore, the emergence of new multidisciplinary treatment approaches is a necessary development. Currently, an increasingly compelling body of research evidence has emerged linking low-grade inflammation state to GDM (5). The immune activation in state of low-grade inflammation is known to decrease β cell function and promote insulin resistance in T2DM (6).

Among these immune activation biomarkers, chemotactic cytokines (chemokines) network plays an important role in the pathogenesis of GDM. Preclinical research has classified chemokines into four subfamilies, namely C, CC, CXC, and CX3C, which are identified by position and presence of the cysteine residue (conserved near the N terminus) (7). Evidence indicates they play an important role in both the immune system and maternal-fetal interface during physiological and pathological pregnancies, which interacts with a group of 7-transmembrane G protein-coupled receptor (GPCRs) (8, 9). Specifically, chemokines network is concerned with the interactions among numerous immune activation factors during pregnancy, including delicate chorus between immune coordination and cellular migration, maintenance of the maternal-fetal interface of feto-maternal tolerance, and attraction of immune cells to the sites of ongoing inflammation (9–11). Considering their role in immune coordination and orchestration of the precise spatio-temporal organization of immune cells, chemokines are prime candidates for linking physiological and pathological pregnancies inflammation, and orchestrating pathogenesis of GDM inflammatory crosstalk.

Although the preceding evidence points to a systemic role of the state of chemokines network in the pathology pregnancies inflammation, what is not known is which chemokines are up-regulated or down-regulated in GDM and their potential role in the pathogenesis of GDM (11–13). Moreover, it is notoriously difficult to identify a complete nomenclature coverage of chemokines, especially from previous older studies because the chemokines nomenclature has not been uniform (14). To date, more than 50 chemokines acting on 23 discrete receptors have been discovered (15). However, existing studies of chemokines in GDM are frequently underpowered, have mostly disparate methodologies, and often have conflicting state of chemokines (16–18). Therefore, we aimed to select historical and current chemokines potentially involved in the pathogenesis of GDM through extensive retrieval structure terms utilizing as many variant chemokines nomenclatures as possible. Then, we compared the concentrations of the circulating maternal chemokines, and placenta or adipose tissue chemokines between patients with GDM and healthy controls using high-quality meta-analytical techniques. We also explored sources of heterogeneity between studies with disparate methodologies, and improved statistical power using subgroup meta-analysis.

## Methods

### Search Strategy and Selection Criteria

This protocol was registered with the International Prospective Register of Systematic Reviews (PROSPERO: CRD42019148305). In addition, this systematic review and meta-analysis was performed in accordance with the Preferred Reporting Items for Systematic Reviews and Meta-Analyses (PRISMA) guidelines and the Cochrane Handbook (19).

A comprehensive search for articles on chemokines and GDM, published in any language, was conducted systematically on August 1st, 2021 in the following databases: Web of Science, Embase, Cochrane Library, and PubMed. Non-English language studies were translated to English. The complete well-designed search strategy is listed in the **Appendix 1**. This was designed by professional librarians who made it as comprehensive as possible, while using truncation and wildcards. Boolean operators were also used in the search strategy to allow for variant historical and current names of chemokines.

Two independent reviewers (XP and AK) grouped relevant eligible studies based on title and abstract screening. Inclusion criteria were defined as: (1) subjects met GDM criteria and reported the method for diagnosing GDM; (2) blood, placenta or adipose tissue samples for chemokines measurement were collected from GDM and healthy controls; (3) the mean and standard deviation (SD) of chemokine were reported, or these data could be acquired by contacting the relevant authors. Exclusion criteria were defined as: (1) letters, reviews, interventional clinical trials, case reports or comments; (2) *in vitro* studies; (3) GDM patients with auto immune and inflammatory disease; and those whose chemokines concentrations were influenced by anti-inflammatory or immunomodulatory drugs for chemokines. Disagreements on eligibility of studies were settled by involving a third reviewer (AL).

### Data Extraction

All eligible studies were stored in a database established by EpiData (version 3.0). Duplicates were removed using EndNote (version X9.1). The following data were extracted from each eligible study by two independent reviewers (SW and AK) using a custom data extraction template. Disagreements about data extraction were settled by consensus. Thus, extracted data included: (1) the title, year of publication and the first author’s name; (2) the country; (3) sample size; (4) ethnicity; (5) GDM cases characteristics such as Body Mass Index (BMI), age, systolic blood pressure (SBP), diastolic blood pressure (DBP), fasting plasma glucose (FPG), 2h postprandial blood glucose, insulin, insulin resistance index (HOMA-IR), hemoglobin A1c (HbA1c), low-density lipoprotein (LDL), high-density lipoprotein (HDL), cholesterol, and triglycerides; (6) method of chemokine measurement; and (7) sample material of chemokine and mean ± SD of chemokine concentrations of GDM cases and the comparison group. Furthermore, information about the risk of bias and quality of eligible studies was extracted according to the Newcastle-Ottawa Quality Assessment Scale (NOS) (20).

### Statistical Analysis

To quantitatively summarize the available data, the effect size of chemokines concentrations on GDM for each study was calculated using Cohen’s d as the weighted standardized mean difference (SMD) in chemokines concentrations between GDM cases and the controls (21). Then, the weighted SMDs and their corresponding 95% confidence intervals (CIs) were pooled in a meta-analysis using a random effects inverse variance model (22, 23). Heterogeneity was assessed using the Cochran’s Q statistic test and quantified using the I² statistic, which measures the proportion of total variability between studies. Thus, the I² values of 25% indicate low heterogeneity, 50% moderate heterogeneity and 75% high heterogeneity (24, 25). Sensitivity analysis was a method that measures how the impact of uncertainties of subgroups can lead to uncertainties on the output variables. It was conducted using the Leave-one-out Method. Subgroup meta-analyses were conducted to explore sources of heterogeneity with respect to the following subgroups based on sample and study characteristics: ethnicity, age, BMI and sample material of chemokine. Furthermore, the between-study probability of publication bias was assessed using the degree of symmetry of funnel plots and the Egger’s Linear Regression Test (26). The package, meta, from R (version 3.5.0) was used for this meta-analysis. The significance level for all statistical tests was set at the 0.05, and all tests were two-sided.

## Results

### Literature Search

**Figure 1:**
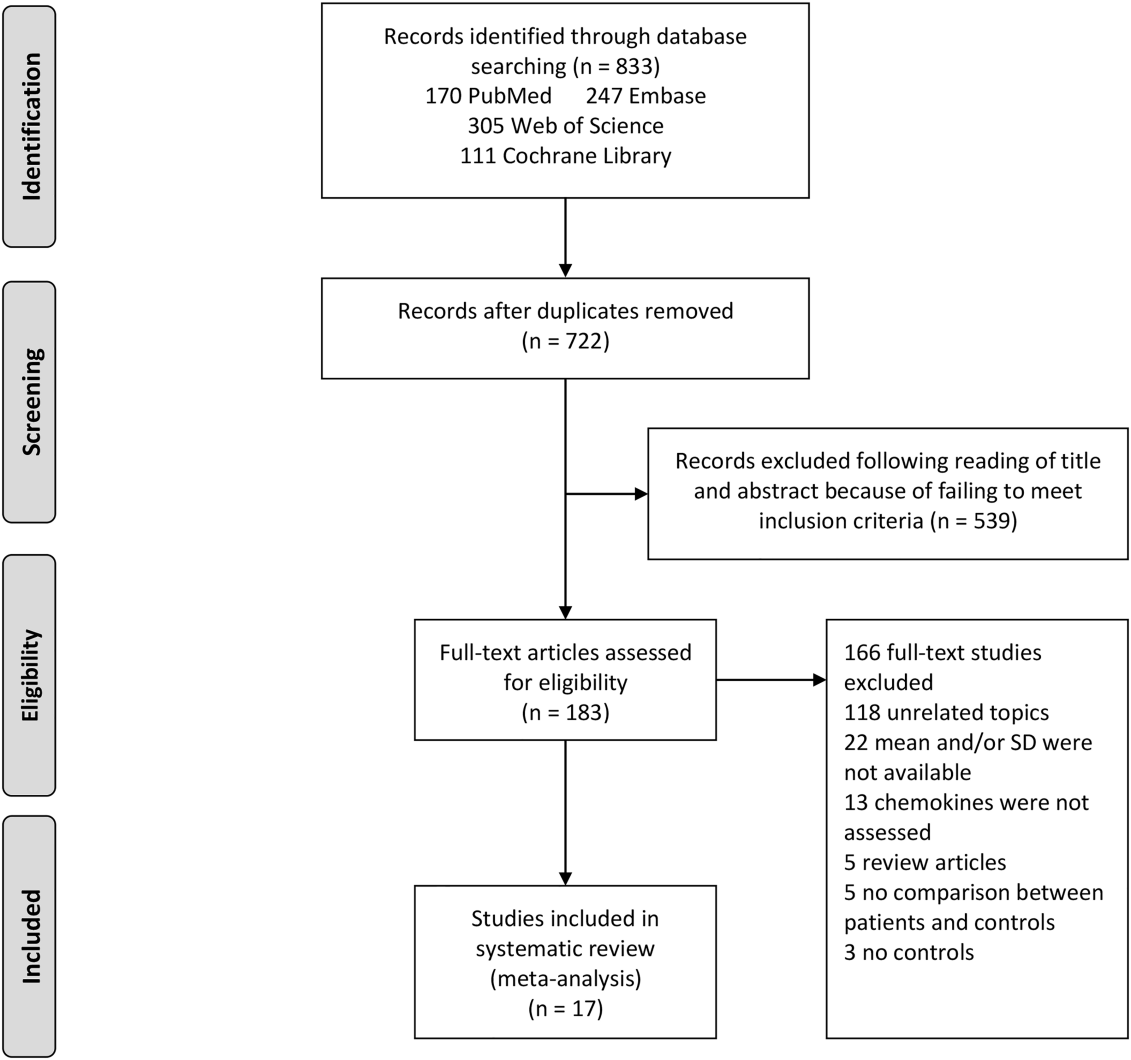
Study selection flow chart. A flow chart demonstrating the selection process of articles included in the analysis as well as in the qualitative summary.

The utilization of the systematic search of four electronic databases yielded a total of 833 studies. After excluding duplicate studies, 722 abstracts were reviewed, of which 539 were excluded. Therefore, the full texts of 183 articles were screened and this led to 17 articles included in this meta-analysis. A flow diagram describing the process of selecting eligible studies is shown in **Figure 1**.

### Characteristics of Eligible Studies

**Table 1** summarizes the characteristics and quality of eligible studies. Among the 17 included studies, eleven studies used maternal blood samples, three studies used cord blood samples, seven studies used placenta samples, while three studies used other samples. Also, in terms of the material of chemokines, 13 studies used the protein, while six studies employed the mRNA. Seven studies used Enzyme-Linked Immunosorbent Assay (ELISA) to determine chemokines, six studies employed the polymerase chain reaction (PCR), while four studies used other methods. The average BMI of GDM patients was 28.28 ± 3.83, and the average age was 31.94 ± 2.34. Ten studies were of high quality, whereas seven were of moderate quality, and the NOS scores varied between 5 and 8. Moreover, anthropometric and clinical phenomics characteristics of included studies are described in the **Appendix 2** and **3**, including SBP, DBP, FPG, 2h Post prandial blood glucose, cholesterol, triglycerides, LDL-C, HDL-C, Insulin, HOMA-IR and HbA1c.

**Table 1:** Characteristics of the studies included for the meta-analysis.

Study		Material	Sample	Country	BMI	Age	Methods	NOS
Chueca 2019	(27)	mRNA	Amnion	Spain	28.00 ± 5.03	36.00 ± 4.06	qPCR	7
Darakhshan 2019	(28)	Protein	Maternal blood	Iran	29.70 ± 1.65	29.60 ± 1.21	ELISA	6
Ebert 2013	(29)	Protein	Maternal blood	Germany	24.50 ± 6.60	31.00 ± 7.50	ELISA	8
Hara 2016	(17)	Protein	Maternal blood/Cord blood/Placenta (villi/extravilli)	Brazil	28.66 ± 4.60	29.55 ± 6.55	CBA	7
Jin 2017	(30)	mRNA	Placenta/Omental adipose tissues	China	28.56	30.13	qPCR	5
Kapustin 2020	(31)	Protein	Maternal blood	Russia	28.80	34.45	ELISA	6
Keckstein 2020	(32)	Protein	EVT/SCT	Germany	28.13 ± 6.96	32.83 ± 4.56	IHC/IF	7
Lappas 2004	(18)	Protein	Maternal blood	Australia	25.70 ± 1.60	35.30 ± 1.60	ELISA	6
Lekva 2017	(33)	Protein/mRNA	Maternal blood/PBMC	Norway	27.80 ± 5.70	33.10 ± 3.70	EIA/RT-qPCR	7
Li 2020	(34)	Protein	Maternal blood/Cord blood	China	28.31 ± 4.63	33.59 ± 5.15	UV	8
Mrizak 2013	(35)	mRNA	Placenta	Tunisia	24.90 ± 2.90	29.50	qPCR	8
Murthy 2018	(16)	Protein	Maternal blood	India	25.70	27.60	ELISA	5
Pan 2021	(36)	mRNA	Placenta	USA	26.65 ± 5.73	33.17 ± 4.65	RT-qPCR	6
Saucedo 2021	(37)	Protein	Maternal blood	Mexico	34.80	32.00	MI	7
Stirm 2018	(38)	Protein/mRNA	Cord blood/Placenta	Germany	30.60 ± 5.60	34.00 ± 4.00	Bio-Plex/qPCR	6
Tang 2021	(39)	Protein	Maternal blood	China	21.32 ± 2.59	32.00	ELISA	8
Zhang 2017	(40)	Protein	Maternal blood/Placenta	China	38.68 ± 9.50	29.13 ± 3.65	ELISA	7

NOS, Newcastle-Ottawa Scale; IHC/IF, immunohistochemistry and immunofluorescence double staining; RT-qPCR, Quantitative reverse transcription polymerase chain reaction; Bio-Plex, Bio-Plex Human Cytokine Assays; qPCR, Real-time polymerase chain reaction/quantitative polymerase chain reaction; BMI, Body Mass Index; PBMC, Plasma protein and peripheral blood mononuclear cells; EIA, Enzyme immunoassay; UV, Ultraviolet spectrophotometry; CBA, Cytometric bead assay; PCR, Polymerase Chain Reaction; MI, Multiplex immunoassay; ELISA, enzyme-linked immunosorbent assay; NR, not report; USA, The United States of America; UK, United Kingdom.

**Appendix 4** systematically summarizes the classification of chemokines and their receptors that may link chemokines to the pathogenesis of GDM, including forty-six chemokines and eighteen chemokine receptors. In addition, **Appendix 5** summarizes the distribution of the cell type of chemokine receptors that may link chemokines to the pathogenesis of GDM in immune microenvironment such as dendric cells, monocytes, macrophages, natural killer cells, Th2 cells, Th17 cells, Treg cells, basophils, CD4 and CD8 T cells.

### Main Outcomes

**Figure 2:**
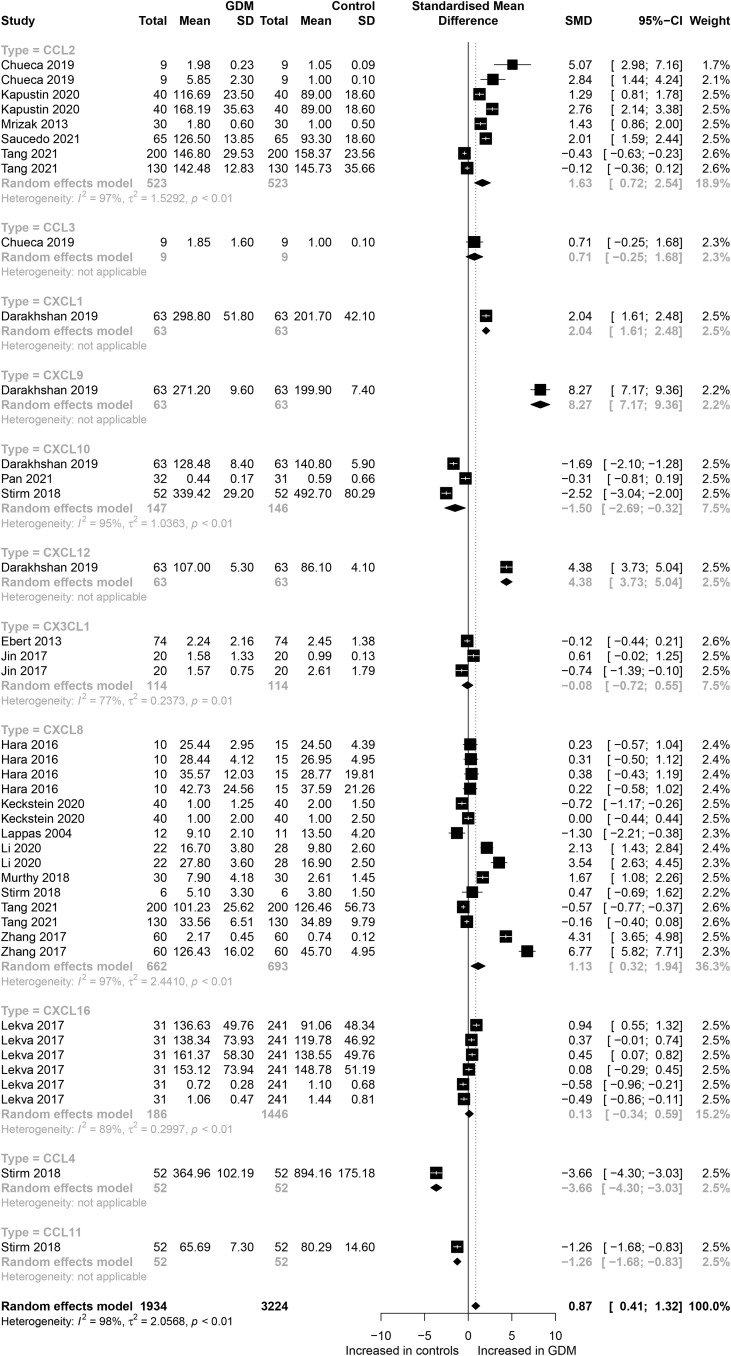
Forest plot of chemokines between GDM patients and controls. Study effect sizes of chemokines differences between GDM and controls. Each data marker represents a study, and the size of the data marker is proportional to the total number of individuals in that study. The summary effect size for each chemokines is denoted by a diamond; GDM, Gestational diabetes mellitus; SMD, standardized mean difference.

The eligible studies included nine different chemokines comparisons involving 5,158 participants (1,934 GDM patients and 3,224 controls). The forest plots of specific chemokines are shown in **Figure 2**. Considering all chemokines involved, chemokines concentrations were significantly higher in the GDM patients than in the controls (SMD=0.87; 95% CI: 0.41; 1.32). However, significantly high heterogeneity was observed in the included studies (*I²=*98%). Similarly, for CCL2 chemokines reported in five studies that included 523 GDM patients and 523 controls, the concentrations of CCL2 were significantly higher in the GDM patients than in the controls (SMD=1.63; 95% CI: 0.72; 2.54). Nevertheless, significant heterogeneity was observed in the included studies (*I²=*97%). For CCL4, the concentrations of CCL4 were significantly lower in the GDM patients than in the controls (SMD=-3.66; 95% CI: -4.30; -3.03). For CCL11, the concentrations of CCL11 were significantly lower in the GDM patients than in the controls (SMD= -1.26; 95% CI: -1.68; -0.83). There was no significant difference in the concentrations of CCL3 between the GDM patients and the controls.

**Figure 3:**
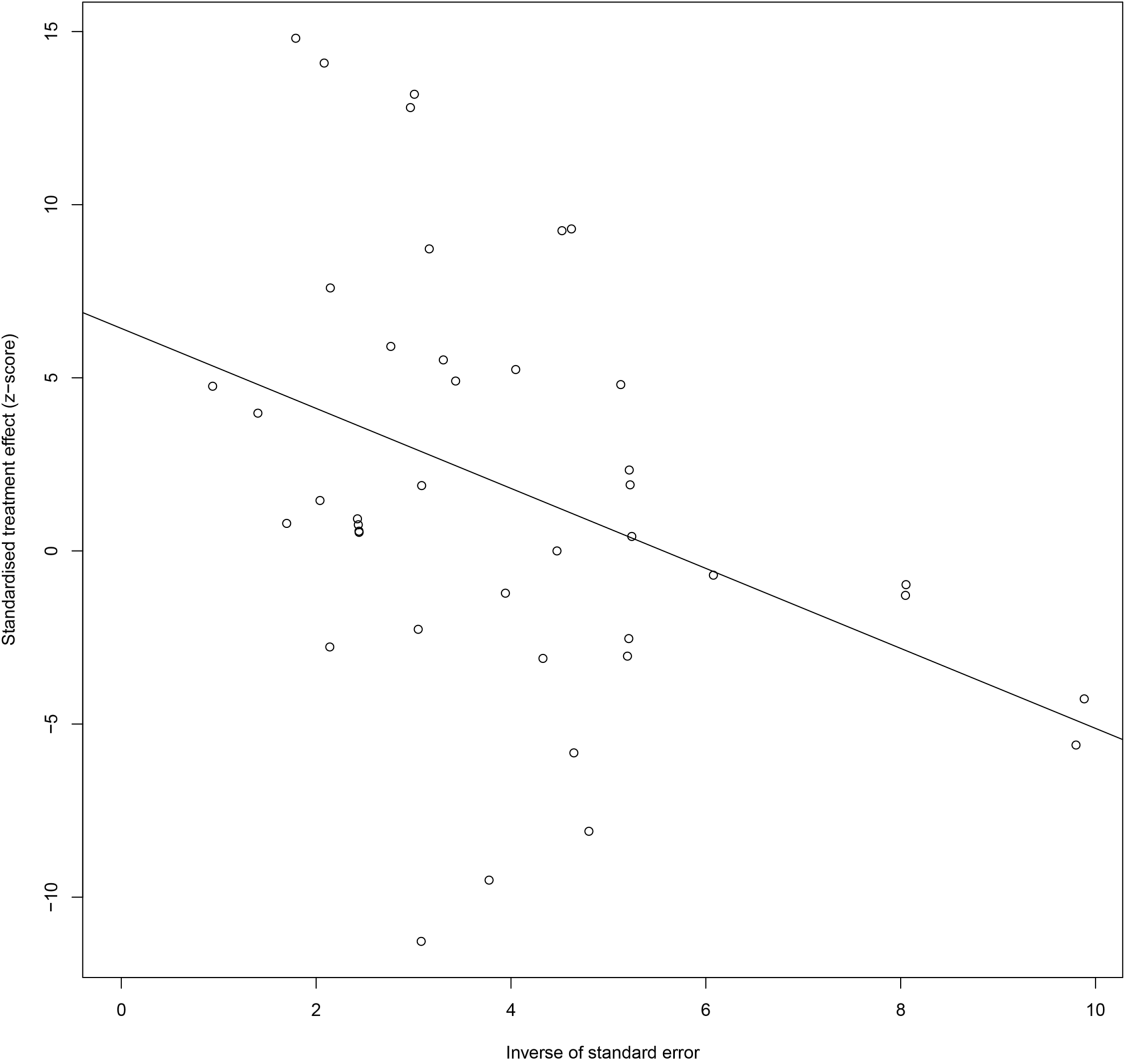
Egger funnel plots of GDM patients compared to controls. GDM patients chemokine and chemokines receptor compared to patients with controls, t = 2.03, p-value = 0.061. Egger funnel plots to assess publication bias. Plots show study size as a function of effect size for studies included in the meta-analysis. The dots represent each study. GDM, Gestational diabetes mellitus.

Also, for CXC chemokines, CXCL1 concentrations were significantly higher in the GDM patients than in the controls (SMD=2.04; 95% CI: 1.61; 2.48); CXCL8 concentrations were significantly higher in the GDM patients than in the controls (SMD=1.13; 95% CI: 0.32; 1.94); CXCL9 concentrations were significantly higher in the GDM patients compared with the controls (SMD=8.27; 95% CI: 7.17; 9.36); CXCL10 concentrations were significantly lower in the GDM patients than in the controls (SMD=-1.50; 95% CI: -2.69; -0.32); CXCL12 concentrations were significantly higher in the GDM patients compared with the controls (SMD=4.38; 95% CI: 3.73; 5.04). There was no significant difference between the GDM patients and the controls in the concentrations of CXCL16 and CX3CL1. The Egger’s Linear Regression tests and funnel plots revealed low probability of potential publication bias (**Figure 3**). Sensitivity analysis indicated that any single eligible study influenced little change in the effect size of chemokines on GDM patients, suggesting that the meta-analysis was stable and not driven by any single study.

### Subgroup Analysis

**Table 2** shows the results of subgroup analysis. Noteworthy, subgroup analysis in this study was divided into laboratory and clinical parts. First, subgroup analyses were conducted to determine the effect of material of chemokines, sample type and assay methods on concentrations of chemokines. These laboratory characteristics were considered for subgroup analyses in view of their well-known effects on chemokines and their pre-clinical implications. Second, subgroup analyses were performed to determine the effect of age, ethnicity, and BMI on concentrations of chemokines. Similarly, these were considered for subgroup analyses following their well-known effects on chemokines and their clinical implications.

**Table 2:** Subgroup analysis of chemokine between GDM participants and controls.

Subgroup		SMD	95% CI		Heterogeneity	
					Q	I²
**Material**	mRNA	0.59	-0.06	1.24	90.99	90.10%
	Protein	**0.91**	0.37	1.46	1562.68	98.10%
**Sample**	Maternal blood	**1.21**	0.63	1.80	1015.76	98.00%
	Cord blood	-0.74	-2.66	1.17	196.45	98.00%
	Placenta	0.96	-0.14	2.05	223.31	96.40%
	Other	0.66	-0.24	1.56	53.92	90.70%
**Age Methods**	≥30	0.23	-0.16	0.61	693.91	96.00%
	<30	**2.34**	0.87	3.81	687.51	98.40%
	PCR	0.59	-0.06	1.24	90.99	90.10%
	ELISA	**1.45**	0.79	2.11	1105.88	98.40%
	Other	0.04	-1.02	1.10	436.51	97.50%
**BMI**	≥28	**1.41**	0.50	2.32	1393.98	98.20%
	<28	0.05	-0.23	0.34	154.46	90.90%
**Ethnicity**	Caucasian	**0.67**	0.07	1.26	1050.66	97.30%
	Mongoloid	**1.45**	0.61	2.29	515.35	98.30%

Subgroup analyses are performed to compare the concentration of chemokines and chemokines receptors between the GDM and the controls. Heterogeneity was quantified using I^2^ and its significance was tested using the Q statistics. GDM, Gestational diabetes mellitus; NR, not report; SMD, standardized mean difference. Bold showed significant difference (P < 0.05).

Regarding sample of chemokines, only maternal blood chemokines (SMD=1.21; 95% CI: 0.63; 1.80) indicated significantly higher concentrations of chemokines in the GDM patients than in the controls. There was no significant difference in cord blood, placenta or other types of samples. Also, for material type, only protein (SMD=0.91; 95% CI: 0.37; 1.46) indicated significantly higher concentrations of chemokines in the GDM patients than in the controls. Studies of mRNA did not reveal a significant difference. Referring to assay methods for chemokines, the ELISA method (SMD=1.45; 95% CI: 0.79; 2.11). Studies of PCR or other types of methods did not reveal a significant difference.

Considering age of the GDM patients, subgroup meta-analysis showed that concentrations of chemokines were increased significantly in the GDM patients with advanced maternal age (SMD=1.41; 95% CI: 0.50; 2.32). Studies of non-advanced maternal age did not reveal a significant difference. Subgroup meta-analysis according to ethnicity showed that both the Caucasian (SMD= 0.67; 95% CI: 0.07; 1.26) and the Mongoloid (SMD=1.45; 95% CI: 0.61; 2.29) had significantly higher concentrations of chemokines in the GDM patients compared with controls. The effect size in relation to the Mongoloid ethnicity was significantly higher than that of the Caucasian ethnicity. Finally, with regard to BMI, subgroup meta-analysis showed that concentrations of chemokines were increased in overweight/obese patients with GDM (SMD=1.41; 95% CI: 0.50; 2.32). However, there was no significant difference in the concentrations of chemokines between the non-overweight/obese GDM patients and the controls.

## Discussion

This is currently the first meta-analysis and systematic review of chemokine ligands and receptors markers in GDM. It found that concentrations of some chemokines were higher in patients with GDM than in controls, while other chemokines had lower concentrations in patients with GDM than in controls. Specifically, the concentrations of the following chemokines were significantly higher in patients with GDM than in controls: CCL2, CXCL1, CXCL8, CXCL9, and CXCL12. On the other hand, the concentrations of the chemokines, CCL4, CCL11 and CXCL10, were significantly lower in patients with GDM than in controls. These results suggest that some chemokines may link immune microenvironment to the pathogenesis of GDM. Therefore, it is imperative to illustrate the unique role of chemokines in the immune microenvironment in order to examine their implications in the pathogenesis, clinical practice and therapeutic targets of GDM.

### Pathogenesis Implications

With regard to the pathogenesis implications, the interactions between chemokines concentrations and maternal immune microenvironment antagonize the release of syncytiotrophoblast debris, which contributes to placental oxidative stress and systemic inflammation (41). Furthermore, the overnutrition and embryo-maternal interactions (the balance between immune suppression and tolerance) during pregnancy can be the major causative factor for the process that probably causes and perpetuates a state of low-grade inflammation, which is common in several pregnancy complications, such as preeclampsia, preterm labor, GDM, and autoimmune diseases (42). These mechanisms of inflammatory responses during pregnancy may link chemokines to the pathogenesis of GDM. In addition, chronic and systemic state of low-grade and sterile inflammation might explain the association between GDM and pathologic state, in which the immune imbalance between pro-inflammatory and anti-inflammatory chemokines has a key role in metabolic abnormalities, glucolipotoxicity, oxidative stress, and tissue-specific insulin resistance (IR) (43).

Specifically, pancreatic islets, peri-pancreatic adipose tissue and immune cells constitute the immune microenvironment of islets. Thus, glucolipotoxicity occurs when the immune microenvironment of islets is exposed to an early damage by genetic or environmental factors, such as overnutrition in the induction of accumulation of elevated levels of glucose/lipids. Consequently, glucolipotoxicity induces sustained activation of various pro-inflammatory and metabolic pathways, and then starts to secrete numerous chemokines (42, 43). The effect of chemokines provokes the immune microenvironment of islets in two aspects. First, chemokines and their receptors are decisively involved in interfering with insulin signaling transcriptional mediated molecular and pro-inflammatory pathways. Moreover, chemokines are one of the major causative factors of the activation of the Adenosine 5-Monophosphate-Activated Protein Kinase (AMPK) Pathway. They also lead to the activation of nuclear factor-kappaB (NF-κB/IκBα) transcriptional mediated molecular, which stimulates a pro-inflammatory condition and blocks the activation of insulin signaling receptors of β cell (44). Meanwhile, chronic exposure to chemokines stimulates the activation of reactive oxygen species/reactive nitrogen species (ROS/RNS) signaling proteins, which is ultimately involved in the endoplasmic reticulum (ER) stress, DNA damage and β cell failure (45).

**Figure 4:**
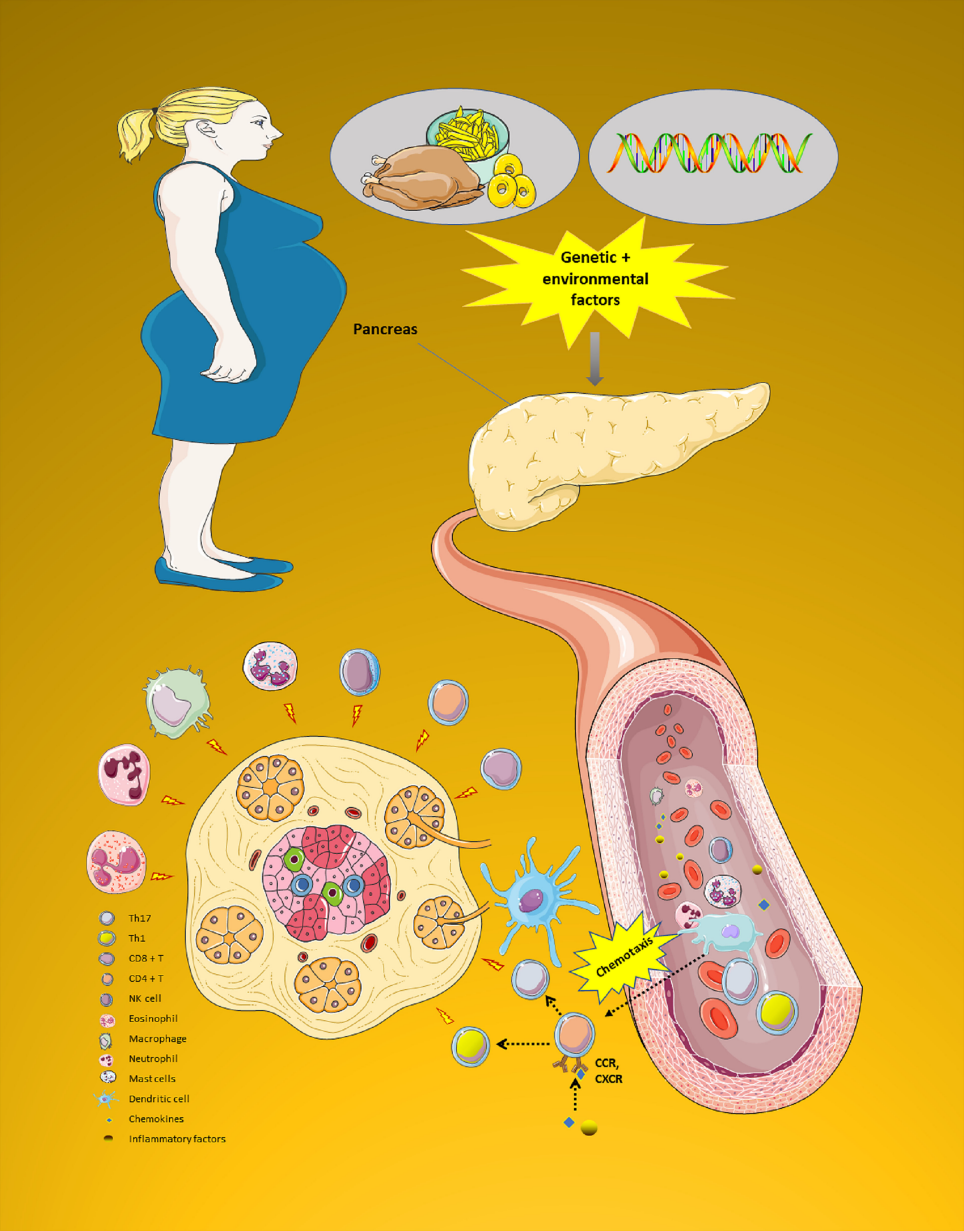
The complicated chemokines network in the pancreatic islets’ microenvironment of GDM. The chemokine system plays a variety of roles in the pancreatic islets microenvironment of GDM. First, pancreatic islets are exposed to an early damage by genetic or environmental factors. Then, chemokines can also cause a variety of immune cells to enter the pancreatic islets site to play the role of immune attack. All these processes impact endoplasmic reticulum stress, leading to a reduction in the ability to secrete insulin. Moreover, GDM progression is characterized by progressive secretion of pro-inflammatory chemokines/cytokines caused by β cell damage. Due to this process, various immune cell types (i.e., CD8+T cell, CD4+T cell (Th1 cell and Th17 cell), NK cell, eosinophil, macrophage, neutrophil, mast cells, and dendritic cell) are recruited in the pancreatic tissue. These immune cells further release more innate inflammatory cytokines, which contribute to a rapid increase β cell death. GDM, Gestational diabetes mellitus; NK, Natural killer. (Drawn by AK).

Second, chemokines can also lead to the activation of various vasculature or circulating immune cells that enter into the immune microenvironment of islets site (7). A number of these immune cells (i.e., CD8+T cell, CD4+T cell (Th1 cell and Th17 cell), Natural Killer (NK) cell, eosinophil, macrophage, neutrophil, mast cells, and dendritic cell) are recruited in the immune microenvironment to further release more pro-inflammatory cytokines (i.e., IL-1β、IL-6、CRP and IL-1β), which provoke the pancreatic islet’s apoptosis and β cell damage by immune attack (14, 15). **Figure 4** shows the complicated chemokines network in the pancreatic islets’ microenvironment of GDM.

### Clinical Implications

The findings of subgroup meta-analysis point to a potential clinical implication in the use of chemokines as biomarkers of GDM. Moreover, the potential role of chemokines as biomarkers is in relation to laboratory detection of biomarkers and clinical characteristics of patients.

In this regard, there were significant differences in the concentrations of chemokines between maternal blood, cord blood and placenta, suggesting that high concentrations of chemokines in maternal circulation cannot enter the fetus through the placental barrier (46). High concentrations of chemokines could be associated with epigenetic changes with DNA methylation including the genes involved in the pathways of immune and inflammatory response, cell growth and death regulation and nervous system development, which increased the risk of future development of T2DM, CAD, obesity, and hypertension in the offspring from mothers with GDM (4, 47).

Furthermore, there were significant differences in the concentrations of chemokines between ELISA, PCR and other types of methods, suggesting that the concentrations of ELISA may have high sensitivity (48). Moreover, considering that a protein is more stable than mRNA, ELISA kit is more convenient in clinical detection (49). Therefore, we recommend that in future clinical and laboratory studies, ELISA samples should be used to detect chemokines in order to obtain more stable results.

Subgroup analysis showed that different ethnicities and ages significantly affect the concentration of chemokines in GDM patients. Subgroup analysis revealed that age significantly affected chemokine circulating concentration in GDM patients. Previous study has shown that the placental functions, including senescence-associated secretory-phenotype production and immune-cell accumulation, gradually decrease in a maternal age-dependent manner, resulting in a higher rate of GDM (50). These results are consistent with age related inflammation kinetics studies, which suggest that patients gradually lose the ability to control excessive inflammatory response with the increase of age. Similarly, previous studies showed that the allele frequencies of single nucleotide polymorphisms of chemokines and their receptor genes were different among different ethnicities (51, 52). Accordingly, this study showed that Mongoloid had a higher effect value than Caucasian. Therefore, we speculate that the genotype-driven treatments in the clinical course of GDM may be a unique window of stratification and individualized pathophysiology-based therapies of GDM patients.

Furthermore, the concentrations of chemokines in this study differed significantly only in GDM patients with BMI≥28. This is consistent with previous studies, which suggested that obesity may contribute to the development and progression of GDM. Therefore, it is reasonable to speculate that this may be due to the glucolipotoxicity in obese GDM patients, which further destroys the homeostasis of immune system and chemokines. Further, experimental studies on molecular mechanisms have shown that adipocyte death can activate macrophages and mediate related inflammation and systemic IR (53). Adipocyte death can lead to the formation of peroxynitrite (ONOO-) (54). Among them, ONOO- further induces related inflammation and systemic IR, mediated by oxidative stress products ROS/RNS (55).

### Therapeutic Implications

The development of anti-inflammatory drugs that address placental oxidative stress and systemic state of low-grade inflammation-lowering CCL2, CXCL1, CXCL8, CXCL9 and CXCL10 might be of potential value in GDM treatment. The CCL2 is one of the critical pro-inflammatory chemokines that belongs to the CC chemokine family. Other names of CCL2 include small inducible cytokine A2 (SCYA2), monocyte chemoattractant and activating factor (MCAF) or monocyte chemoattractant protein (MCP)-1. The CCL2 and its cognate receptor CCR2 play a key role in regulating infiltration and migration of Th1 cells, basophil, NK cells, monocytes, and macrophages in the immune microenvironment of islets. Recent research has shown that higher concentrations of CCL2 in serum correlated with early IR, carbohydrate metabolism disorder, obesity development, and preeclampsia development risk (12, 56). CCL2, CCL4, and CCL11 are considered pro-inflammatory chemokines. CCL2 is a CC chemokine with specificity for CCR2 receptors. CCL4 and CCL11 are exerting a wide range of activities through the CCR5 receptors (57). This is consistent with our findings and may suggest that CCL2 is a critical biomarker of GDM as well as a target for therapeutic intervention.

In GDM patients, with the dysfunction of β cells, even before β cells are widely damaged, CCL4 concentrations may rise ahead of time. While resulting in β cells death and early islet graft loss, inflammatory stimuli with a CD40-CD40L interaction could induce the secretion of CCL4 through the Raf/MEK/ERK and NF-κB pathways in pancreatic islets (58). Therefore, CCL4 concentrations may be caused by the initial inflammatory damage of islet β cells.

As a CXC chemokine, the function of CXCL8 is the induction of chemotaxis in its target cells, like neutrophil granulocytes, basophils, T-cells, and adipocytes. There are many receptors capable of binding CXCL8. Those with the most affinity to CXCL8 are receptors CXCR1 and CXCR2 (59). Studies have shown that CXCL8 secreted by adipocytes may be related to complications such as GDM, which are the accumulation of excess accumulation of intra-abdominal fat (60). Increasing evidence suggests that the intra-abdominal fat accumulation is closely related to decreased insulin sensitivity and increased GDM pathophysiology (61). The results of our study suggest that GDM patients have higher concentrations of CXCL8, which is consistent with the findings of previous research. CXCL8 may mediate the downregulation of adiponectin in obesity. Adiponectin can prevent the impairment of insulin signaling, so CXCL8 may plays a crucial and causal role in obesity-linked IR and GDM (62).

Additionally, both the CXCL9 and CXCL10 are the critical pro-inflammatory and angiostasis chemokines that belong to the CXC chemokine family. These chemokines and their cognate receptor CXCR3 play a key role in regulating infiltration and migration of basophils, Th1 cells, CD8+T cells, NK cells, and Treg cells into pancreatic tissue. A clinical study also showed that the concentrations of CXCL10 in T2DM patients were higher than that in the controls (63). Moreover, both CXCL9 and CXCL10 have been attributed to several roles in IR pathogenesis, such as influencing islet β cell mass and decreasing β cell viability. Similarly, an animal experiment showed that both CXCL9 and CXCL10 played a key role in regulating infiltration and migration of basophils and NK cells into the immune microenvironment of islets, which impaired insulin secretion and function in mice with genetic deletion of CXCR3 (64). Besides, CXCL10 leads to the activation of c-Jun N-terminal kinase (JNK), and protein kinase B (Akt) *via* chemokine receptor CXCR3 (65, 66). The CXCL10 also induces the cleavage of p21-activated protein kinase 2 (PAK-2) and triggering β cell oxidative stress and destruction (63, 66). However, this is inconsistent with our findings, possibly due to the small number of studies included in the meta-analyses. Future studies should therefore further explore the relationship between CXCL10 and GDM.

The CXCL1 is one of the critical pro-inflammatory angiogenesis that belongs to the CXC chemokine family. The CXCL1 and its cognate receptor CXCR2 play a key role in regulating infiltration and migration of neutrophils, monocytes, mast cells, basophils, dendric cells, and NK cells in the immune microenvironment of islets. A recent clinical study has also shown higher concentrations of CXCL1 in GDM patients than in controls. Thus, CXCL1 may be involved in the pathogenesis of GDM through endothelial damage and TNF-α production (67). Also, CXCL1 and its cognate receptor CXCR4, and CXCR7 play a key role in lymphopoiesis and promote angiogenesis in the immune microenvironment of islets. Moreover, CXCL12 is one of the critical factors for angiogenesis that belongs to the CXC chemokine family. A sib-pair study also showed that CXCL12 genetic polymorphisms were associated with T2DM (68). Therefore, CXCL12 might probably be involved in the GDM pathophysiologically due to its association with angiogenesis. This is consistent with a previous study, which reported a close relationship between angiogeneic chemokines, such as CXCL1 and CXCL12, and endothelium damage and IR (69).

Furthermore, the CXCL16 is one of the pro-inflammatory single-pass type I membrane protein that belongs to the CXC chemokine family. The CXCL16 and its cognate receptor CXCR6 play a key role in regulating infiltration and migration of Th1 cells, Th17 cells, and NK cells in the immune microenvironment of islets. A clinical study showed elevated concentrations of CXCL16 in T2DM, coronary artery disease (CAD), and GDM in early pregnancy and after 5 years (70, 71). Moreover, higher levels of CXCL16 correlated with LDL-C and apoli-poprotein B (apoB) (72). Also, CXCL16 acts as a scavenger receptor on macrophages, hence promoting oxidation and internalization of LDL-C, which may play an important role in the inflammatory response due to lipid accumulation. However, this is inconsistent with our findings, perhaps due to the small number of studies included in the meta-analysis. Future studies need to further explore the relationship between CXCL16 and GDM.

The main strength of this study is that it has summarized the effect of as many chemokines as possible on GDM, which was achieved through a comprehensive search strategy that used multiple variant names of chemokines in a range of databases following PRISMA guidelines. Nevertheless, our analysis used a limited number of studies reporting the effects of the chemokines, CCL3, CCL4, CCL11 and CXCL12, on GDM, which may lead to biased or heterogeneous results. Furthermore, this study was limited by the lack of original research data on confounding variables at the individual level, such as smoking status, alcohol consumption, physical activity, blood pressure, or any combination of these variables. Therefore, these were not taken into account in the meta-analysis. Also, this study used the original data of case-control studies, implying that the findings do not make any causal inference. Therefore, more fundamental mechanistic research and longitudinal study designs, taking into account confounding variables, are essential if we are to truly understand the distinct pathways involved in chemokine biology and the pathophysiological mechanisms of GDM.

## Conclusions

In this meta-analysis, several chemokines (CCL2, CCL4, CCL11, CXCL1, CXCL8, CXCL9, CXCL10 and CXCL12) were found to be altered in patients with GDM. The contribution of our study was to summarize latest developments in chemokines molecular mechanisms leading to the pathophysiology of GDM development. This allowed future challenges and opportunities for individualized clinical and therapeutic implications of GDM in relation to chemokines to be discussed.

## Data Availability Statement

The original contributions presented in the study are included in the article/**Supplementary Material**. Further inquiries can be directed to the corresponding author.

## Author Contributions

XP and AL contributed to the study design, while SW and AK contributed to the data collection. Statistical analyses and interpretation of results were performed by XP and AK, whereas JM, XP and SW drafted the manuscript and edited the language. All the authors participated in the critical revisions, and approved the final version of the manuscript.

## Funding

The research is financially supported by Hunan Provincial Key Laboratory of Clinical Epidemiology and the Hunan Provincial Key Research and Development Program (2018SK2065), China.

## Conflict of Interest

The authors declare that the research was conducted in the absence of any commercial or financial relationships that could be construed as a potential conflict of interest.

## Publisher’s Note

All claims expressed in this article are solely those of the authors and do not necessarily represent those of their affiliated organizations, or those of the publisher, the editors and the reviewers. Any product that may be evaluated in this article, or claim that may be made by its manufacturer, is not guaranteed or endorsed by the publisher.
