# Supplementary material for: Chemokines in Gestational Diabetes Mellitus
Source: Front Immunol. 2022 Feb 8;13:705852. doi: 10.3389/fimmu.2022.705852 (PMC8860907; doi:10.3389/fimmu.2022.705852)
Supplement: Supplementary file 1 [file DataSheet_1.doc]

**Supplementary appendix**

**Contents**

**Appendix 1**: Electronic search strategies

**Appendix 2:** Anthropometric and clinical phenomics characteristics of included studies

**Appendix 3:** Clinical phenomics characteristics of included studies

**Appendix 4:** The classification of chemokines and their receptors.

**Appendix 5**: The distribution—cell type of chemokines receptors.

**Appendices References**

**Appendix 1**: Electronic search strategies

| **Search** | **Query** |
| --- | --- |
| 1 | chemokine* |
| 2 | ccl1 or ccl2 or ccl3 or ccl4 or ccl5 or ccl6 or ccl7 or ccl8 or ccl9 or ccl10 or ccl11 or ccl12 or ccl13 or ccl14 or ccl15 or ccl16 or ccl17 or ccl18 or ccl19 or ccl20 or ccl21 or ccl22 or ccl23 or ccl24 or ccl25 or ccl26 or ccl27 or ccl28 |
| 3 | cxcl1 or cxcl2 or cxcl3 or cxcl4 or cxcl5 or cxcl6 or cxcl7 or cxcl8 or cxcl9 or cxcl10 or cxcl11 or cxcl12 or cxcl13 or cxcl14 or cxcl15 or cxcl16 or cxcl17 |
| 4 | xcl1 or xcl2 |
| 5 | cx3cl1 |
| 6 | ccl or cxcl or xcl or cx3cl |
| 7 | scya1 or scya2 or scya3 or scya4 or scya5 or scya6 or scya7 or scya8 or scya9 or scya10 or scya11 or scya12 or scya13 or scya14 or scya15 or scya16 or scya17 or scya18 or scya19 or scya20 or scya21 or scya22 or scya23 or scya24 or scya25 or scya26 or scya27 or scya28 |
| 8 | scyb1 or scyb2 or scyb3 or scyb4 or scyb5 or scyb6 or scyb7 or scyb8 or scyb9 or scyb10 or scyb11 or scyb12 or scyb13 or scyb14 or scyb15 or scyb16 or scyb17 |
| 9 | scyc1 or scyc2 |
| 10 | sycd1 |
| 11 | scya or scyb or scyc or scyd |
| 12 | chemokine receptor* |
| 13 | ccr1 or ccr2 or ccr2b or ccr3 or ccr4 or ccr5 or ccr6 or ccr7 or ccr8 or ccr9 or ccr10 |
| 14 | cxcr1 or cxcr2 or cxcr3 or cxcr3b or cxcr4 or cxcr5 or cxcr6 or cxcr7 |
| 15 | xcr1 |
| 16 | cx3cr1 |
| 17 | ccr or cxcr or xcr or cx3cr |
| 18 | chemotactic cytokine* or chemokine* |
| 19 | i-309 or i309 or tca-3 or tca3 or sise |
| 20 | IL-8 or GCP-2 or CXCR1 or NAP-2 or ENA-78 or GROα or GROβ or GROγ or PF4 or IP-10 or MIG or I-TAC or SDF-1 or BCA-1 or SR-PSOX or BRAK or MCP-1 or MCP-4 or CCR2 or MCP-3 or MCP-2 or MIP-1β or MIP-1α or CCR5 or RANTES or MPIF-1 or HCC-1 or HCC-2 or HCC-4 or Eotaxin or Eotaxin-2 or Eotaxin-3 or TARC or CCR4 or MDC or MIP-3α or ELC or CCR7 or SLC or I-309 or TECK or CTACK or MEC or PARC or Lymphotactin or XCR1 or SCM-1β or Fractalkine or Chemerin |
| 21 | S1 OR S2 OR S3 OR S4 OR S5 OR S6 OR S7 OR S8 OR S9 OR S10 OR S11 OR S12 OR S13 OR S14 OR S15 OR S16 OR S17 OR S18 OR S19 OR S20 |
| 22 | Diabetes Mellitus, Gestational OR Diabetes, Gestational OR Mellitus, Gestational OR Diabetic, Gestational |
| 23 | Diabete, Gestational OR Glycuresis, Gestational OR Diabetics, Gestational OR Gestational diabetes OR Gestational diabetes mellitus OR GDM OR Diabetes, Pregnancy-Induced OR Diabetes, Pregnancy Induced OR Pregnancy-Induced Diabetes |
| 24 | S22 OR S23 |
| 25 | S21 AND S24 |

**305 of Web of Science**

TOPIC: (Diabetes Mellitus, Gestational OR Diabetes, Gestational OR Mellitus, Gestational OR Diabetic, Gestational OR Diabete, Gestational OR Glycuresis, Gestational OR Diabetics, Gestational OR Gestational diabetes OR Gestational diabetes mellitus OR GDM OR Diabetes, Pregnancy-Induced OR Diabetes, Pregnancy Induced OR Pregnancy-Induced Diabetes) AND TOPIC: (chemokine* or ccl1 or ccl2 or ccl3 or ccl4 or ccl5 or ccl6 or ccl7 or ccl8 or ccl9 or ccl10 or ccl11 or ccl12 or ccl13 or ccl14 or ccl15 or ccl16 or ccl17 or ccl18 or ccl19 or ccl20 or ccl21 or ccl22 or ccl23 or ccl24 or ccl25 or ccl26 or ccl27 or ccl28 or cxcl1 or cxcl2 or cxcl3 or cxcl4 or cxcl5 or cxcl6 or cxcl7 or cxcl8 or cxcl9 or cxcl10 or cxcl11 or cxcl12 or cxcl13 or cxcl14 or cxcl15 or cxcl16 or cxcl17 or xcl1 or xcl2 or cx3cl1 or ccl or cxcl or xcl or cx3cl or scya1 or scya2 or scya3 or scya4 or scya5 or scya6 or scya7 or scya8 or scya9 or scya10 or scya11 or scya12 or scya13 or scya14 or scya15 or scya16 or scya17 or scya18 or scya19 or scya20 or scya21 or scya22 or scya23 or scya24 or scya25 or scya26 or scya27 or scya28 or scyb1 or scyb2 or scyb3 or scyb4 or scyb5 or scyb6 or scyb7 or scyb8 or scyb9 or scyb10 or scyb11 or scyb12 or scyb13 or scyb14 or scyb15 or scyb16 or scyb17 or scyc1 or scyc2 or sycd1 or scya or scyb or scyc or scyd or chemokine receptor* or ccr1 or ccr2 or ccr2b or ccr3 or ccr4 or ccr5 or ccr6 or ccr7 or ccr8 or ccr9 or ccr10 or cxcr1 or cxcr2 or cxcr3 or cxcr3b or cxcr4 or cxcr5 or cxcr6 or cxcr7 or xcr1 or cx3cr1 or ccr or cxcr or xcr or cx3cr or chemotactic cytokine* or chemokine* or i-309 or i309 or tca-3 or tca3 or sise or IL-8 or GCP-2 or CXCR1 or NAP-2 or ENA-78 or GROα or GROβ or GROγ or PF4 or IP-10 or MIG or I-TAC or SDF-1 or BCA-1 or SR-PSOX or BRAK or MCP-1 or MCP-4 or CCR2 or MCP-3 or MCP-2 or MIP-1β or MIP-1α or CCR5 or RANTES or MPIF-1 or HCC-1 or HCC-2 or HCC-4 or Eotaxin or Eotaxin-2 or Eotaxin-3 or TARC or CCR4 or MDC or MIP-3α or ELC or CCR7 or SLC or I-309 or TECK or CTACK or MEC or PARC or Lymphotactin or XCR1 or SCM-1β or Fractalkine or Chemerin or Limphotactin or SCM-1 or C-10 or Mrp-1 or MIP-1γ or MRP2 or Eotaxin-1 or MCP-5 or Leukotactin-1 or MIP-5 or LEC or NCC-4 or MTN1 or MIP-4 or AMAC1 or ELC or MIP-3β or LARC or MIP-3 or 6Ckine or MPIF-2 or Eotaxin-2 or MIP-4α or CTAK or MGSA or MIP-2α or MIP-2β or PF-4 or BLC or Lungkine or SRPSOX)

**247 of Embase**

('diabetes mellitus, gestational':ti, ab, kw OR 'diabetes, gestational':ti, ab, kw OR 'mellitus, gestational':ti, ab, kw OR 'diabetic, gestational':ti, ab, kw OR 'diabete, gestational':ti, ab, kw OR 'glycuresis, gestational':ti, ab, kw OR 'diabetics, gestational':ti, ab, kw OR 'gestational diabetes':ti, ab, kw OR 'gestational diabetes mellitus':ti, ab, kw OR gdm:ti, ab, kw OR 'diabetes, pregnancy-induced':ti, ab, kw OR 'diabetes, pregnancy induced':ti, ab, kw OR 'pregnancy-induced diabetes':ti, ab, kw) AND (ccl1:ti, ab, kw OR ccl2:ti, ab, kw OR ccl3:ti, ab, kw OR ccl4:ti, ab, kw OR ccl5:ti, ab, kw OR ccl6:ti, ab, kw OR ccl7:ti, ab, kw OR ccl8:ti, ab, kw OR ccl9:ti, ab, kw OR ccl10:ti, ab, kw OR ccl11:ti, ab, kw OR ccl12:ti, ab, kw OR ccl13:ti, ab, kw OR ccl14:ti, ab, kw OR ccl15:ti, ab, kw OR ccl16:ti, ab, kw OR ccl17:ti, ab, kw OR ccl18:ti, ab, kw OR ccl19:ti, ab, kw OR ccl20:ti, ab, kw OR ccl21:ti, ab, kw OR ccl22:ti, ab, kw OR ccl23:ti, ab, kw OR ccl24:ti, ab, kw OR ccl25:ti, ab, kw OR ccl26:ti, ab, kw OR ccl27:ti, ab, kw OR ccl28:ti, ab, kw OR cxcl1:ti, ab, kw OR cxcl2:ti, ab, kw OR cxcl3:ti, ab, kw OR cxcl4:ti, ab, kw OR cxcl5:ti, ab, kw OR cxcl6:ti, ab, kw OR cxcl7:ti, ab, kw OR cxcl8:ti, ab, kw OR cxcl9:ti, ab, kw OR cxcl10:ti, ab, kw OR cxcl11:ti, ab, kw OR cxcl12:ti, ab, kw OR cxcl13:ti, ab, kw OR cxcl14:ti, ab, kw OR cxcl15:ti, ab, kw OR cxcl16:ti, ab, kw OR cxcl17:ti, ab, kw OR xcl1:ti, ab, kw OR xcl2:ti, ab, kw OR cx3cl1:ti, ab, kw OR ccl:ti, ab, kw OR cxcl:ti, ab, kw OR xcl:ti, ab, kw OR cx3cl:ti, ab, kw OR scya1:ti, ab, kw OR scya2:ti, ab, kw OR scya3:ti, ab, kw OR scya4:ti, ab, kw OR scya5:ti, ab, kw OR scya6:ti, ab, kw OR scya7:ti, ab, kw OR scya8:ti, ab, kw OR scya9:ti, ab, kw OR scya10:ti, ab, kw OR scya11:ti, ab, kw OR scya12:ti, ab, kw OR scya13:ti, ab, kw OR scya14:ti, ab, kw OR scya15:ti, ab, kw OR scya16:ti, ab, kw OR scya17:ti, ab, kw OR scya18:ti, ab, kw OR scya19:ti, ab, kw OR scya20:ti, ab, kw OR scya21:ti, ab, kw OR scya22:ti, ab, kw OR scya23:ti, ab, kw OR scya24:ti, ab, kw OR scya25:ti, ab, kw OR scya26:ti, ab, kw OR scya27:ti, ab, kw OR scya28:ti, ab, kw OR scyb1:ti, ab, kw OR scyb2:ti, ab, kw OR scyb3:ti, ab, kw OR scyb4:ti, ab, kw OR scyb5:ti, ab, kw OR scyb6:ti, ab, kw OR scyb7:ti, ab, kw OR scyb8:ti, ab, kw OR scyb9:ti, ab, kw OR scyb10:ti, ab, kw OR scyb11:ti, ab, kw OR scyb12:ti, ab, kw OR scyb13:ti, ab, kw OR scyb14:ti, ab, kw OR scyb15:ti, ab, kw OR scyb16:ti, ab, kw OR scyb17:ti, ab, kw OR scyc1:ti, ab, kw OR scyc2:ti, ab, kw OR sycd1:ti, ab, kw OR scya:ti, ab, kw OR scyb:ti, ab, kw OR scyc:ti, ab, kw OR scyd:ti, ab, kw OR 'chemokine receptor*':ti, ab, kw OR ccr1:ti, ab, kw OR ccr2b:ti, ab, kw OR ccr3:ti, ab, kw OR ccr6:ti, ab, kw OR ccr8:ti, ab, kw OR ccr9:ti, ab, kw OR ccr10:ti, ab, kw OR cxcr2:ti, ab, kw OR cxcr3:ti, ab, kw OR cxcr3b:ti, ab, kw OR cxcr4:ti, ab, kw OR cxcr5:ti, ab, kw OR cxcr6:ti, ab, kw OR cxcr7:ti, ab, kw OR cx3cr1:ti, ab, kw OR ccr:ti, ab, kw OR cxcr:ti, ab, kw OR xcr:ti, ab, kw OR cx3cr:ti, ab, kw OR 'chemotactic cytokine*':ti, ab, kw OR chemokine*:ti, ab, kw OR i309:ti, ab, kw OR 'tca 3':ti, ab, kw OR tca3:ti, ab, kw OR sise:ti, ab, kw OR 'il 8':ti, ab, kw OR 'gcp 2':ti, ab, kw OR cxcr1:ti, ab, kw OR 'nap 2':ti, ab, kw OR 'ena 78':ti, ab, kw OR groα:ti, ab, kw OR groβ:ti, ab, kw OR groγ:ti, ab, kw OR pf4:ti, ab, kw OR 'ip 10':ti, ab, kw OR mig:ti, ab, kw OR 'i tac':ti, ab, kw OR 'sdf 1':ti, ab, kw OR 'bca 1':ti, ab, kw OR 'sr psox':ti, ab, kw OR brak:ti, ab, kw OR 'mcp 1':ti, ab, kw OR 'mcp 4':ti, ab, kw OR ccr2:ti, ab, kw OR 'mcp 3':ti, ab, kw OR 'mcp 2':ti, ab, kw OR 'mip 1β':ti, ab, kw OR 'mip 1α':ti, ab, kw OR ccr5:ti, ab, kw OR rantes:ti, ab, kw OR 'mpif 1':ti, ab, kw OR 'hcc 1':ti, ab, kw OR 'hcc 2':ti, ab, kw OR 'hcc 4':ti, ab, kw OR eotaxin:ti, ab, kw OR 'eotaxin 3':ti, ab, kw OR tarc:ti, ab, kw OR ccr4:ti, ab, kw OR mdc:ti, ab, kw OR 'mip 3α':ti, ab, kw OR ccr7:ti, ab, kw OR slc:ti, ab, kw OR 'i 309':ti, ab, kw OR teck:ti, ab, kw OR ctack:ti, ab, kw OR mec:ti, ab, kw OR parc:ti, ab, kw OR lymphotactin:ti, ab, kw OR xcr1:ti, ab, kw OR 'scm 1β':ti, ab, kw OR fractalkine:ti, ab, kw OR chemerin:ti, ab, kw OR limphotactin:ti, ab, kw OR 'scm 1':ti, ab, kw OR 'c 10':ti, ab, kw OR 'mrp 1':ti, ab, kw OR 'mip 1γ':ti, ab, kw OR mrp2:ti, ab, kw OR 'eotaxin 1':ti, ab, kw OR 'mcp 5':ti, ab, kw OR 'leukotactin 1':ti, ab, kw OR 'mip 5':ti, ab, kw OR lec:ti, ab, kw OR 'ncc 4':ti, ab, kw OR mtn1:ti, ab, kw OR 'mip 4':ti, ab, kw OR amac1:ti, ab, kw OR elc:ti, ab, kw OR 'mip 3β':ti, ab, kw OR larc:ti, ab, kw OR 'mip 3':ti, ab, kw OR 6ckine:ti, ab, kw OR 'mpif 2':ti, ab, kw OR 'eotaxin 2':ti, ab, kw OR 'mip 4α':ti, ab, kw OR ctak:ti, ab, kw OR mgsa:ti, ab, kw OR 'mip 2α':ti, ab, kw OR 'mip 2β':ti, ab, kw OR 'pf 4':ti, ab, kw OR blc:ti, ab, kw OR lungkine:ti, ab, kw OR srpsox:ti, ab, kw)

**170 of Pubmed**

(Diabetes Mellitus, Gestational[Title/Abstract] OR Diabetes, Gestational[Title/Abstract] OR Mellitus, Gestational[Title/Abstract] OR Diabetic, Gestational[Title/Abstract] OR Diabete, Gestational[Title/Abstract] OR Glycuresis, Gestational[Title/Abstract] OR Diabetics, Gestational[Title/Abstract] OR Gestational diabetes[Title/Abstract] OR Gestational diabetes mellitus[Title/Abstract] OR GDM[Title/Abstract] OR Diabetes, Pregnancy-Induced[Title/Abstract] OR Diabetes, Pregnancy Induced[Title/Abstract] OR Pregnancy-Induced Diabetes[Title/Abstract]) AND (chemokine*[Title/Abstract] OR ccl1[Title/Abstract] OR ccl2[Title/Abstract] OR ccl3[Title/Abstract] OR ccl4[Title/Abstract] OR ccl5[Title/Abstract] OR ccl6[Title/Abstract] OR ccl7[Title/Abstract] OR ccl8[Title/Abstract] OR ccl9[Title/Abstract] OR ccl10[Title/Abstract] OR ccl11[Title/Abstract] OR ccl12[Title/Abstract] OR ccl13[Title/Abstract] OR ccl14[Title/Abstract] OR ccl15[Title/Abstract] OR ccl16[Title/Abstract] OR ccl17[Title/Abstract] OR ccl18[Title/Abstract] OR ccl19[Title/Abstract] OR ccl20[Title/Abstract] OR ccl21[Title/Abstract] OR ccl22[Title/Abstract] OR ccl23[Title/Abstract] OR ccl24[Title/Abstract] OR ccl25[Title/Abstract] OR ccl26[Title/Abstract] OR ccl27[Title/Abstract] OR ccl28[Title/Abstract] OR cxcl1[Title/Abstract] OR cxcl2[Title/Abstract] OR cxcl3[Title/Abstract] OR cxcl4[Title/Abstract] OR cxcl5[Title/Abstract] OR cxcl6[Title/Abstract] OR cxcl7[Title/Abstract] OR cxcl8[Title/Abstract] OR cxcl9[Title/Abstract] OR cxcl10[Title/Abstract] OR cxcl11[Title/Abstract] OR cxcl12[Title/Abstract] OR cxcl13[Title/Abstract] OR cxcl14[Title/Abstract] OR cxcl15[Title/Abstract] OR cxcl16[Title/Abstract] OR cxcl17[Title/Abstract] OR xcl1[Title/Abstract] OR xcl2[Title/Abstract] OR cx3cl1[Title/Abstract] OR ccl[Title/Abstract] OR cxcl[Title/Abstract] OR xcl[Title/Abstract] OR cx3cl[Title/Abstract] OR scya1[Title/Abstract] OR scya2[Title/Abstract] OR scya3[Title/Abstract] OR scya4[Title/Abstract] OR scya5[Title/Abstract] OR scya6[Title/Abstract] OR scya7[Title/Abstract] OR scya8[Title/Abstract] OR scya9[Title/Abstract] OR scya10[Title/Abstract] OR scya11[Title/Abstract] OR scya12[Title/Abstract] OR scya13[Title/Abstract] OR scya14[Title/Abstract] OR scya15[Title/Abstract] OR scya16[Title/Abstract] OR scya17[Title/Abstract] OR scya18[Title/Abstract] OR scya19[Title/Abstract] OR scya20[Title/Abstract] OR scya21[Title/Abstract] OR scya22[Title/Abstract] OR scya23[Title/Abstract] OR scya24[Title/Abstract] OR scya25[Title/Abstract] OR scya26[Title/Abstract] OR scya27[Title/Abstract] OR scya28[Title/Abstract] OR scyb1[Title/Abstract] OR scyb2[Title/Abstract] OR scyb3[Title/Abstract] OR scyb4[Title/Abstract] OR scyb5[Title/Abstract] OR scyb6[Title/Abstract] OR scyb7[Title/Abstract] OR scyb8[Title/Abstract] OR scyb9[Title/Abstract] OR scyb10[Title/Abstract] OR scyb11[Title/Abstract] OR scyb12[Title/Abstract] OR scyb13[Title/Abstract] OR scyb14[Title/Abstract] OR scyb15[Title/Abstract] OR scyb16[Title/Abstract] OR scyb17[Title/Abstract] OR scyc1[Title/Abstract] OR scyc2[Title/Abstract] OR sycd1[Title/Abstract] OR scya[Title/Abstract] OR scyb[Title/Abstract] OR scyc[Title/Abstract] OR scyd[Title/Abstract] OR chemokine receptor*[Title/Abstract] OR ccr1[Title/Abstract] OR ccr2[Title/Abstract] OR ccr2b[Title/Abstract] OR ccr3[Title/Abstract] OR ccr4[Title/Abstract] OR ccr5[Title/Abstract] OR ccr6[Title/Abstract] OR ccr7[Title/Abstract] OR ccr8[Title/Abstract] OR ccr9[Title/Abstract] OR ccr10[Title/Abstract] OR cxcr1[Title/Abstract] OR cxcr2[Title/Abstract] OR cxcr3[Title/Abstract] OR cxcr3b[Title/Abstract] OR cxcr4[Title/Abstract] OR cxcr5[Title/Abstract] OR cxcr6[Title/Abstract] OR cxcr7[Title/Abstract] OR xcr1[Title/Abstract] OR cx3cr1[Title/Abstract] OR ccr[Title/Abstract] OR cxcr[Title/Abstract] OR xcr[Title/Abstract] OR cx3cr[Title/Abstract] OR chemotactic cytokine*[Title/Abstract] OR chemokine*[Title/Abstract] OR i-309[Title/Abstract] OR i309[Title/Abstract] OR tca-3[Title/Abstract] OR tca3[Title/Abstract] OR sise[Title/Abstract] OR IL-8[Title/Abstract] OR GCP-2[Title/Abstract] OR CXCR1[Title/Abstract] OR NAP-2[Title/Abstract] OR ENA-78[Title/Abstract] OR GROα[Title/Abstract] OR GROβ[Title/Abstract] OR GROγ[Title/Abstract] OR PF4[Title/Abstract] OR IP-10[Title/Abstract] OR MIG[Title/Abstract] OR I-TAC[Title/Abstract] OR SDF-1[Title/Abstract] OR BCA-1[Title/Abstract] OR SR-PSOX[Title/Abstract] OR BRAK[Title/Abstract] OR MCP-1[Title/Abstract] OR MCP-4[Title/Abstract] OR CCR2[Title/Abstract] OR MCP-3[Title/Abstract] OR MCP-2[Title/Abstract] OR MIP-1β[Title/Abstract] OR MIP-1α[Title/Abstract] OR CCR5[Title/Abstract] OR RANTES[Title/Abstract] OR MPIF-1[Title/Abstract] OR HCC-1[Title/Abstract] OR HCC-2[Title/Abstract] OR HCC-4[Title/Abstract] OR Eotaxin[Title/Abstract] OR Eotaxin-2[Title/Abstract] OR Eotaxin-3[Title/Abstract] OR TARC[Title/Abstract] OR CCR4[Title/Abstract] OR MDC[Title/Abstract] OR MIP-3α[Title/Abstract] OR ELC[Title/Abstract] OR CCR7[Title/Abstract] OR SLC[Title/Abstract] OR I-309[Title/Abstract] OR TECK[Title/Abstract] OR CTACK[Title/Abstract] OR MEC[Title/Abstract] OR PARC[Title/Abstract] OR Lymphotactin[Title/Abstract] OR XCR1[Title/Abstract] OR SCM-1β[Title/Abstract] OR Fractalkine[Title/Abstract] OR Chemerin[Title/Abstract] OR Limphotactin[Title/Abstract] OR SCM-1[Title/Abstract] OR C-10[Title/Abstract] OR Mrp-1[Title/Abstract] OR MIP-1γ[Title/Abstract] OR MRP2[Title/Abstract] OR Eotaxin-1[Title/Abstract] OR MCP-5[Title/Abstract] OR Leukotactin-1[Title/Abstract] OR MIP-5[Title/Abstract] OR LEC[Title/Abstract] OR NCC-4[Title/Abstract] OR MTN1[Title/Abstract] OR MIP-4[Title/Abstract] OR AMAC1[Title/Abstract] OR ELC[Title/Abstract] OR MIP-3β[Title/Abstract] OR LARC[Title/Abstract] OR MIP-3[Title/Abstract] OR 6Ckine[Title/Abstract] OR MPIF-2[Title/Abstract] OR Eotaxin-2[Title/Abstract] OR MIP-4α[Title/Abstract] OR CTAK[Title/Abstract] OR MGSA[Title/Abstract] OR MIP-2α[Title/Abstract] OR MIP-2β[Title/Abstract] OR PF-4[Title/Abstract] OR BLC[Title/Abstract] OR Lungkine[Title/Abstract] OR SRPSOX[Title/Abstract])

**111 of Cochrane Library**

chemokine* or ccl1 or ccl2 or ccl3 or ccl4 or ccl5 or ccl6 or ccl7 or ccl8 or ccl9 or ccl10 or ccl11 or ccl12 or ccl13 or ccl14 or ccl15 or ccl16 or ccl17 or ccl18 or ccl19 or ccl20 or ccl21 or ccl22 or ccl23 or ccl24 or ccl25 or ccl26 or ccl27 or ccl28 or cxcl1 or cxcl2 or cxcl3 or cxcl4 or cxcl5 or cxcl6 or cxcl7 or cxcl8 or cxcl9 or cxcl10 or cxcl11 or cxcl12 or cxcl13 or cxcl14 or cxcl15 or cxcl16 or cxcl17 or xcl1 or xcl2 or cx3cl1 or ccl or cxcl or xcl or cx3cl or scya1 or scya2 or scya3 or scya4 or scya5 or scya6 or scya7 or scya8 or scya9 or scya10 or scya11 or scya12 or scya13 or scya14 or scya15 or scya16 or scya17 or scya18 or scya19 or scya20 or scya21 or scya22 or scya23 or scya24 or scya25 or scya26 or scya27 or scya28 or scyb1 or scyb2 or scyb3 or scyb4 or scyb5 or scyb6 or scyb7 or scyb8 or scyb9 or scyb10 or scyb11 or scyb12 or scyb13 or scyb14 or scyb15 or scyb16 or scyb17 or scyc1 or scyc2 or sycd1 or scya or scyb or scyc or scyd or chemokine receptor* or ccr1 or ccr2 or ccr2b or ccr3 or ccr4 or ccr5 or ccr6 or ccr7 or ccr8 or ccr9 or ccr10 or cxcr1 or cxcr2 or cxcr3 or cxcr3b or cxcr4 or cxcr5 or cxcr6 or cxcr7 or xcr1 or cx3cr1 or ccr or cxcr or xcr or cx3cr or chemotactic cytokine* or chemokine* or i-309 or i309 or tca-3 or tca3 or sise or IL-8 or GCP-2 or CXCR1 or NAP-2 or ENA-78 or GROα or GROβ or GROγ or PF4 or IP-10 or MIG or I-TAC or SDF-1 or BCA-1 or SR-PSOX or BRAK or MCP-1 or MCP-4 or CCR2 or MCP-3 or MCP-2 or MIP-1β or MIP-1α or CCR5 or RANTES or MPIF-1 or HCC-1 or HCC-2 or HCC-4 or Eotaxin or Eotaxin-2 or Eotaxin-3 or TARC or CCR4 or MDC or MIP-3α or ELC or CCR7 or SLC or I-309 or TECK or CTACK or MEC or PARC or Lymphotactin or XCR1 or SCM-1β or Fractalkine or Chemerin or Limphotactin or SCM-1 or C-10 or Mrp-1 or MIP-1γ or MRP2 or Eotaxin-1 or MCP-5 or Leukotactin-1 or MIP-5 or LEC or NCC-4 or MTN1 or MIP-4 or AMAC1 or ELC or MIP-3β or LARC or MIP-3 or 6Ckine or MPIF-2 or Eotaxin-2 or MIP-4α or CTAK or MGSA or MIP-2α or MIP-2β or PF-4 or BLC or Lungkine or SRPSOX in Title Abstract Keyword AND Diabetes Mellitus, Gestational OR Diabetes, Gestational OR Mellitus, Gestational OR Diabetic, Gestational OR Diabete, Gestational OR Glycuresis, Gestational OR Diabetics, Gestational OR Gestational diabetes OR Gestational diabetes mellitus OR GDM OR Diabetes, Pregnancy-Induced OR Diabetes, Pregnancy Induced OR Pregnancy-Induced Diabetes in Title Abstract Keyword

**Appendix 2: Anthropometric and clinical phenomics c**haracteristics of included studies

| **Study** |  | **SBP (mmHg)** | **DBP (mmHg)** | **FPG (mmol/l)** | **2h Post prandial blood glucose (mmol/l)** | **HbA1c (%)** |
| --- | --- | --- | --- | --- | --- | --- |
| Chueca 2019 | [1] | NR | NR | NR | NR | 5.36±0.25 |
| Darakhshan 2019 | [2] | NR | NR | NR | NR | NR |
| Ebert 2013 | [3] | 120.00±20.00 | 73.00±15.00 | 4.50±0.90 | 8.70±2.30 | 5.40±0.60 |
| Hara 2016 | [4] | NR | NR | NR | NR | 5.79±0.89 |
| Jin 2017 | [5] | 113.73 (109.75-119.20) | 71.62 (67.69-78.76) | NR | NR | NR |
| Kapustin 2020 | [6] | NR | NR | NR | NR | 5.80 (5.30-6.30) |
| Keckstein 2020 | [7] | NR | NR | NR | NR | NR |
| Lappas 2004 | [8] | NR | NR | 4.90±0.20 | NR | NR |
| Lekva 2017 | [9] | 110.00 (110.00-120.00) | 70.00 (60.00-70.00) | NR | NR | NR |
| Li 2020 | [10] | NR | NR | 5.04±0.93 | NR | 5.56±0.53 |
| Mrizak 2013 | [11] | NR | NR | 6.80±0.66 | NR | 6.90±0.45 |
| Murthy 2018 | [12] | NR | NR | NR | NR | NR |
| Pan 2021 | [13] | NR | NR | NR | NR | NR |
| Saucedo 2021 | [14] | NR | NR | 4.70 (4.10-5.30) | 9.80 (8.70-10.50) | NR |
| Stirm 2018 | [15] | NR | NR | 4.80±0.50 | 8.40±1.00 | NR |
| Tang 2021 | [16] | 109.83±10.14 | 67.44±0.80 | 4.64±0.53 | 9.07±1.24 | 5.20 (5.00-5.40) |
| Zhang 2017 | [17] | NR | NR | 8.03±1.57 | NR | NR |

NR, not report; FPG, fasting plasma glucose; SBP, systolic blood pressure; DBP, diastolic blood pressure; HbA1c, haemoglobin A1c.

**Appendix 3: C**linical phenomics characteristics of included studies

| **Study** |  | **Cholesterol (mg/dL)** | **Triglycerides (mg/dL)** | **LDL-c (mmol/l)** | **HDL-c (mmol/l)** | **Insulin (μIU/ml)** | **HOMA-IR** |
| --- | --- | --- | --- | --- | --- | --- | --- |
| Chueca 2019 | [1] | 9.78±6.35 | 3.50±7.41 | NR | NR | 18.27±9.59 | 3.98±2.20 |
| Darakhshan 2019 | [2] | 3.94±0.94 | NR | NR | NR | NR | NR |
| Ebert 2013 | [3] | 6.71±1.74 | 2.14±1.31 | 4.05±1.91 | 1.82±0.80 | 7.60±6.70 | 1.99±1.90 |
| Hara 2016 | [4] | NR | NR | NR | NR | 6.65±2.07 | NR |
| Jin 2017 | [5] | NR | NR | NR | NR | 8.55 (7.46-12.75) | 2.01 (1.66-3.09) |
| Kapustin 2020 | [6] | NR | NR | NR | NR | NR | NR |
| Keckstein 2020 | [7] | NR | NR | NR | NR | NR | NR |
| Lappas 2004 | [8] | NR | NR | NR | NR | 12.10±1.60 | NR |
| Lekva 2017 | [9] | NR | NR | 2.62 (2.02-3.09) | NR | NR | NR |
| Li 2020 | [10] | 6.22±1.10 | 3.48±1.63 | 3.01±0.93 | 1.69±0.35 | 9.13±4.78 | NR |
| Mrizak 2013 | [11] | 5.19±0.32 | 2.48±0.15 | 2.16±0.26 | 2.20±0.12 | 10.55±4.80 | NR |
| Murthy 2018 | [12] | NR | NR | NR | NR | NR | NR |
| Pan 2021 | [13] | NR | NR | NR | NR | NR | NR |
| Saucedo 2021 | [14] | 55.80 (49.30-66.00) | 3.40 (2.20-3.90) | 1.4 (0.84-2.1) | 2.40 (1.90-3.00) | 6.40 (4.40-8.70) | 1.7 (1.3-2.5) |
| Stirm 2018 | [15] | NR | NR | NR | NR | NR | NR |
| Tang 2021 | [16] | NR | NR | NR | NR | 6.79 (4.75-10.78) | 1.38 (0.95-2.21) |
| Zhang 2017 | [17] | NR | NR | NR | NR | 3.15±1.08 | 4.89±1.40 |

NR, not report; LDL, low-density lipoprotein; HDL, high-density lipoprotein; TG, triglycerides.

**Appendix 4**: The classification of chemokines and their receptors.

| **Subfamily of chemokine** | **Name of chemokine** | **Other names of chemokine** | **Receptors** |
| --- | --- | --- | --- |
| **C chemokine** | XCL1 | Limphotactin α, SCM-1α | XCR1 |
|  | XCL2 | Limphotactin β, SCM-1β | XCR1 |
| **CC chemokine** | CCL1 | I-309 | CCR8 |
|  | CCL2 | MCP-1 | CCR2 |
|  | CCL3 | MIP-1α | CCR1, CCR5 |
|  | CCL4 | MIP-1β | CCR5, CCR8 |
|  | CCL5 | RANTES | CCR1, CCR3, CCR5 |
|  | CCL6 | C-10, Mrp-1 | CCR1 |
|  | CCL7 | MCP-3 | CCR1, CCR2, CCR3 |
|  | CCL8 | MCP-2 | CCR1, CCR2, CCR3, CCR5, CCR8 |
|  | CCL9 | MIP-1γ, MRP2 | CCR1, CCR3 |
|  | CCL11 | Eotaxin-1 | CCR3, CCR5 |
|  | CCL12 | MCP-5 | CCR2 |
|  | CCL13 | MCP-4 | CCR1, CCR2，CCR3, CCR5 |
|  | CCL14 | HCC-1 | CCR1, CCR5 |
|  | CCL15 | HCC-2, Leukotactin-1, MIP-5 | CCR1, CCR3 |
|  | CCL16 | HCC-4, LEC, NCC-4, MTN1 | CCR1, CCR2, CCR5, CCR8 |
|  | CCL17 | TARC | CCR4 |
|  | CCL18 | MIP-4, AMAC1 | CCR8 |
|  | CCL19 | ELC, MIP-3β | CCR7 |
|  | CCL20 | LARC, MIP-3α | CCR6 |
|  | CCL21 | SLC, 6Ckine | CCR7 |
|  | CCL22 | MDC | CCR4 |
|  | CCL23 | MPIF-1, MIP-3 | CCR1, CCR3 |
|  | CCL24 | MPIF-2, Eotaxin-2 | CCR3 |
|  | CCL25 | TECK | CCR9 |
|  | CCL26 | Eotaxin-3, MIP-4α | CCR3, CX3CR1 |
|  | CCL27 | CTAK | CCR10 |
|  | CCL28 | MEC | CCR3, CCR10 |
| **CXC chemokine** | CXCL1 | GROα, MGSA | CXCR2 |
|  | CXCL2 | GROβ, MIP-2α | CXCR2 |
|  | CXCL3 | GROγ, MIP-2β | CXCR2 |
|  | CXCL4 | PF-4 | CXCR3 |
|  | CXCL5 | ENA-78 | CXCR2 |
|  | CXCL6 | GCP-2 | CXCR1, CXCR2 |
|  | CXCL7 | NAP-2 | CXCR2 |
|  | CXCL8 | IL-8 | CXCR1, CXCR2 |
|  | CXCL9 | Mig | CXCR3 |
|  | CXCL10 | IP-10 | CXCR3 |
|  | CXCL11 | I-TAC | CXCR3, CXCR7 |
|  | CXCL12 | SDF-1 | CXCR4, CXCR7 |
|  | CXCL13 | BCA-1, BLC | CXCR5, CXCR3 |
|  | CXCL14 | BRAK | Unknown |
|  | CXCL15 | Lungkine | Unknown |
|  | CXCL16 | SRPSOX | CXCR6 |
| **CX3C chemokine** | CX3CL1 | Fractalkine | CX3CR1 |

**Appendix 5**: The distribution—cell type of chemokines receptors.

| **Receptors of Chemokine** | **Distribution—Cell Type** |
| --- | --- |
| **CXCR1** | Neutrophils, monocytes, mast cells, basophils, dendric cells, CD8 T cells, natural killer cells |
| **CXCR2** | Neutrophils, monocytes, mast cells, basophils, dendric cells, natural killer cells |
| **CXCR3** | Basophils, Th1 cells, CD8 T cells, natural killer cells, Treg cells |
| **CXCR4** | Widely expressed |
| **CXCR5** | Basophils, CD8 T cells |
| **CXCR6** | Th1 cells, Th17 cells, natural killer cells, plasma cells |
| **CCR2** | Monocytes, macrophages, Th1 cells, basophil, natural killer cells |
| **CCR5** | Dendric cells, monocytes, macrophages, natural killer cells, Th1 cells, TH17 cells, |
| **CCR1** | Neutrophils, monocytes, macrophages, Th1 cells, basophils, dendric cells |
| **CCR3** | Eosinophils, basophils, Th2 cells, mast cells, dendric cells |
| **CCR4** | Th2 cells, Th17 cells, Treg cells, monocytes, basophils, CD4 & CD8 T cells |
| **CCR6** | Th17 cells, natural killer cells, Treg cells |
| **CCR7** | Dendric cells (mature), T cells, basophils |
| **CCR8** | Dendirc cells, monocytes, macrophages, Th2 cells, Treg cells |
| **CCR9** | Basophils, dendric cells |
| **CCR10** | T cells, IgA+ plasma cells |
| **XCR1** | Dendric cells |
| **CX3CR1** | Monocytes, macrophages, Th1 cells, dendric cells, natural killer cells |

**References**

1 Algaba-Chueca F, Maymo-Masip E, Ejarque M, Ballesteros M, Llaurado G, Lopez C, et al. Gestational diabetes impacts fetal precursor cell responses with potential consequences for offspring. Stem Cells Transl Med. 2020;9(3):351-63.

2 Darakhshan S, Fatehi A, Hassanshahi G, Mahmoodi S, Hashemi MS, Karimabad MN. Serum concentration of angiogenic (CXCL1, CXCL12) and angiostasis (CXCL9, CXCL10) CXC chemokines are differentially altered in normal and gestational diabetes mellitus associated pregnancies. Journal of diabetes and metabolic disorders. 2019;18(2):371-78.

3 Ebert T, Hindricks J, Kralisch S, Lossner U, Jessnitzer B, Richter J, et al. Serum levels of fractalkine are associated with markers of insulin resistance in gestational diabetes. Diabetic Medicine. 2014;31(8):1014-17.

4 Hara Cde C, Franca EL, Fagundes DL, de Queiroz AA, Rudge MV, Honorio-Franca AC, et al. Characterization of Natural Killer Cells and Cytokines in Maternal Placenta and Fetus of Diabetic Mothers. Journal of immunology research. 2016;2016:7154524.

5 Jin B, Liu L, Zhang S, Cao X, Xu Y, Wang J, et al. Nuclear Magnetic Resonance-Assisted Metabolic Analysis of Plasma for Mild Gestational Diabetes Mellitus Patients. Metab Syndr Relat Disord. 2017;15(9):439-49.

6 Kapustin RV, Chepanov SV, Babakov VN, Rogovskaya NY, Kopteeva EV, Alekseenkova EN, et al. Maternal serum leptin, adiponectin, resistin and monocyte chemoattractant protein-1 levels in different types of diabetes mellitus. Eur J Obstet Gynecol Reprod Biol. 2020;254:284-91.

7 Keckstein S, Pritz S, Amann N, Meister S, Beyer S, Jegen M, et al. Sex Specific Expression of Interleukin 7, 8 and 15 in Placentas of Women with Gestational Diabetes. Int J Mol Sci. 2020;21(21).

8 Lappas M, Permezel M, Rice GE. Release of proinflammatory cytokines and 8-isoprostane from placenta, adipose tissue, and skeletal muscle from normal pregnant women and women with gestational diabetes mellitus. J Clin Endocrinol Metab. 2004;89(11):5627-33.

9 Lekva T, Michelsen AE, Aukrust P, Paasche Roland MC, Henriksen T, Bollerslev J, et al. CXC chemokine ligand 16 is increased in gestational diabetes mellitus and preeclampsia and associated with lipoproteins in gestational diabetes mellitus at 5 years follow-up. Diabetes and Vascular Disease Research. 2017;14(6):525-33.

10 Li YX, Long DL, Liu J, Qiu D, Wang J, Cheng X, et al. Gestational diabetes mellitus in women increased the risk of neonatal infection via inflammation and autophagy in the placenta. Medicine (Baltimore). 2020;99(40):e22152.

11 Mrizak I, Grissa O, Henault B, Fekih M, Bouslema A, Boumaiza I, et al. Placental infiltration of inflammatory markers in gestational diabetic women. Gen Physiol Biophys. 2014;33(2):169-76.

12 Sudharshana Murthy KA, Bhandiwada A, Chandan SL, Gowda SL, Sindhusree G. Evaluation of Oxidative Stress and Proinflammatory Cytokines in Gestational Diabetes Mellitus and Their Correlation with Pregnancy Outcome. Indian J Endocrinol Metab. 2018;22(1):79-84.

13 Pan X, Jin X, Wang J, Hu Q, Dai B. Placenta inflammation is closely associated with gestational diabetes mellitus. Am J Transl Res. 2021;13(5):4068-79.

14 Saucedo R, Valencia J, Moreno-Gonzalez LE, Pena-Cano MI, Aranda-Martinez A, Garcia Y, et al. Maternal serum adipokines and inflammatory markers at late gestation and newborn weight in mothers with and without gestational diabetes mellitus. Ginekol Pol. 2021.

15 Stirm L, Kovarova M, Perschbacher S, Michlmaier R, Fritsche L, Siegel-Axel D, et al. BMI-Independent Effects of Gestational Diabetes on Human Placenta. J Clin Endocrinol Metab. 2018;103(9):3299-309.

16 Tang M, Luo M, Lu W, Zhang R, Liang W, Gu J, et al. Nerve growth factor is closely related to glucose metabolism, insulin sensitivity and insulin secretion in the second trimester: a case-control study in Chinese. Nutr Metab (Lond). 2020;17(1):98.

17 Zhang J, Chi H, Xiao H, Tian X, Wang Y, Yun X, et al. Interleukin 6 (IL-6) and Tumor Necrosis Factor alpha (TNF-alpha) Single Nucleotide Polymorphisms (SNPs), Inflammation and Metabolism in Gestational Diabetes Mellitus in Inner Mongolia. Medical science monitor : international medical journal of experimental and clinical research. 2017;23:4149-57.
